# Estimated causes of death in Thailand, 2005: implications for health policy

**DOI:** 10.1186/1478-7954-8-14

**Published:** 2010-05-18

**Authors:** Yawarat Porapakkham, Chalapati Rao, Junya Pattaraarchachai, Warangkana Polprasert, Theo Vos, Timothy Adair, Alan D Lopez

**Affiliations:** 1 Ministry of Public Health, Bangkok, Thailand; 2 School of Population Health, University of Queensland, Brisbane, Australia; 3 Department of Community Medicine, Thammasat University, Pathumthani, Thailand; 4 School of Health Services, Thummathirat Open University, Bangkok, Thailand; 5 Health Information Systems Knowledge Hub, University of Queensland, Brisbane, Australia

## Abstract

**Background:**

Almost 400,000 deaths are registered each year in Thailand. Their value for public health policy and planning is greatly diminished by incomplete registration of deaths and by concerns about the quality of cause-of-death information. This arises from misclassification of specified causes of death, particularly in hospitals, as well as from extensive use of ill-defined and vague codes to attribute the underlying cause of death. Detailed investigations of a sample of deaths in and out of hospital were carried out to identify misclassification of causes and thus derive a best estimate of national mortality patterns by age, sex, and cause of death.

**Methods:**

A nationally representative sample of 11,984 deaths in 2005 was selected, and verbal autopsy interviews were conducted for almost 10,000 deaths. Verbal autopsy procedures were validated against 2,558 cases for which medical record review was possible. Misclassification matrices for leading causes of death, including ill-defined causes, were developed separately for deaths inside and outside of hospitals and proportionate mortality distributions constructed. Estimates of mortality undercount were derived from "capture-recapture" methods applied to the 2005-06 Survey of Population Change. Proportionate mortality distributions were applied to this mortality "envelope" and ill-defined causes redistributed according to Global Burden of Disease methods to yield final estimates of mortality levels and patterns in 2005.

**Results:**

Estimated life expectancy in Thailand in 2005 was 68.5 years for males and 75.6 years for females, two years lower than vital registration data suggest. Upon correction, stroke is the leading cause of death in Thailand (10.7%), followed by ischemic heart disease (7.8%) and HIV/AIDS (7.4%). Other leading causes are road traffic accidents (males) and diabetes mellitus (females). In many cases, estimated mortality is at least twice what is estimated in vital registration. Leading causes of death have remained stable since 1999, with the exception of a large decline in HIV/AIDS mortality.

**Conclusions:**

Field research into the accuracy of cause-of-death data can result in substantially different patterns of mortality than suggested by routine death registration. Misclassification errors are likely to have very significant implications for health policy debates. Routine incorporation of validated verbal autopsy methods could significantly improve cause-of-death data quality in Thailand.

## Introduction

A reliable assessment of mortality by age, sex, and cause provides the fundamental evidence to guide health program evaluation and the planning of health services, including prevention programs. The optimal source for population-level data on causes of death by age and sex is complete national vital registration, with medical certification of cause for each registered death [1,2]. In Thailand, death registration is incomplete, but more critically, it suffers from poor quality of recorded causes of death [3,4]. Between 35% and 40% of registered deaths are classified to ill-defined conditions; moreover, there is considerable uncertainty regarding the accuracy of cause-of-death attribution to specific causes [5]. As a result, mortality data from the Thai death registration system cannot be directly used for burden of disease estimation, as is routinely done in countries with higher quality data.

To address this need, a large-scale field study was undertaken to verify registered causes of death in a nationally representative sample of 11,984 deaths that occurred in 2005. This study formed the basis of a broader initiative to develop burden of disease estimates and cost-effectiveness evaluations of major interventions, based on the recommended methodologies reported in the World Health Organization's (WHO) 2002 World Health Report, "Reducing Risks: Promoting Healthy Life." [6]. The application of these methodologies to Thailand represents the first complete national application of the WHO methodology.

The detailed study design, methods, and findings are described in previous articles in this series [4,5,7]. The study was conducted along two broad arms--to estimate mortality patterns for hospital deaths in the study sample and for deaths outside hospitals. The two key objectives of this research were to: understand the likely patterns of misclassification by cause in each setting that result in the high proportion of ill-defined deaths observed in the registration data; and to determine the validity of diagnoses for deaths classified to specific causes. This paper presents national estimates of cause-specific mortality by age and sex for Thailand in 2005, derived from the detailed findings from the two arms of the field study. Appropriate demographic adjustments to allow for gaps in completeness of death registration were applied, as were Global Burden of Disease (GBD) methods to redistribute ill-defined causes to derive the final national mortality estimates [8]. These results provide a comprehensive account of the descriptive epidemiology of mortality in Thailand based on field research, and should serve as primary evidence for burden of disease assessments and cost-effectiveness analyses of intervention options in Thailand.

## Methods

### Estimating all-cause mortality

To estimate cause-specific mortality in a population for which limited data are available, it is important to first develop an estimate of total deaths by age and sex (the so-called "mortality envelope") by adjusting data from the national vital registration database for likely underreporting of deaths. This conforms to the Global Burden of Disease approach whereby the envelope of deaths serves to constrain competing estimates of the numbers of deaths from specific causes [8]. Findings from the 2005-06 Survey of Population Change (SPC) conducted by the Thai National Statistics Office were used to make these adjustments [9]. The SPC is a nationally representative, stratified sample of about 85,000 households covering all provinces (including Bangkok) that is conducted every mid-decade since the mid-1960s. After a baseline population enumeration, four additional rounds were conducted at three-month intervals to identify deaths that occurred in households during the preceding three months. In the event of a death being declared, the household was required to provide proof of the death having been registered at the local or district registration office. On this basis, the National Statistics Office's reported completeness of Thai death registration data for 2005 was estimated to be 95% for deaths at ages above 5 years [9].

Unfortunately, this method is likely to overestimate the completeness of death registration because deaths that occurred but were not reported for whatever reason are omitted from the denominator. Studies that have attempted to match deaths reported in surveys with those reported from routine registration, as in the Kanchanaburi Demographic Surveillance site in Thailand, report lower levels of completeness of about 88% [10]. We have applied this same capture-recapture methodology to the 2005-06 SPC results, matching deaths reported for the study sample with those for the same population in the vital statistics. Of the 1,044 deaths reported in the SPC, 94 were not found in the vital statistics. Conversely, of the 1,236 deaths recorded in vital registration, 286 were not found by the SPC, suggesting an overall level of mortality underregistration of 9.2% (9.0% males, 9.5% females) from application of the Chandrasekar-Deming method [11]. Underregistration of deaths tended to be higher at younger ages (12.8% at ages 5-49), decreasing to 7.7% at ages 50-74 years, and 5.6% at ages 75 and over (Pattama Vapattanawong, personal communication). We applied these age- and sex-specific estimates of incompleteness to adjust the recorded numbers of deaths in 2005 from vital registration. With these adjustments, adult mortality rates increased exponentially with age with the exception of the oldest age group, 85 years and over. To account for undercount of deaths at these ages, we adjusted the observed death rates based on fitting an exponential curve to corrected death rates at ages 40-84 years. This adjustment resulted in the inclusion of an additional 3,026 male deaths and 1,909 female deaths at ages 85 and over.

The SPC also provided estimates of child (under 5) mortality. We compared these with corresponding estimates from other surveys and censuses in Thailand over the past four decades to estimate levels of child mortality prevailing in 2005 by fitting a curve to the observed data [12]. These methods suggest that the undercount of child deaths in vital registration for Thailand is considerably greater than for adults, at approximately 50%.

### Estimating cause-specific mortality

We have prepared estimates of causes of death in two ways. First, we adjusted the original vital registration data for Thailand in 2005 (Table S1 [see Additional file 1]) using the findings from the two field studies [5,7] to estimate the numbers of deaths by age, sex, and cause in Thailand in 2005. These include deaths from ill-defined and other vague causes and reflect the inability of vital registration practices and empirical epidemiological research to correctly specify the cause of all deaths. Second, we have corrected these estimates from adjusted vital registration by redistributing ill-defined and other vague causes of death according to the methods of the GBD study [8,13] In order to achieve comparability with similar estimates from a previous study in Thailand as well as for international comparison, we present our findings according to the standard GBD list of causes [13].

To develop proportionate mortality distributions by age, sex, and cause, we first applied the results of our misclassification studies for deaths in hospitals and outside hospitals in Thailand [5,7]. The corrected proportionate distributions by cause for deaths in hospital were fitted to the total number of reported hospital deaths by age and sex from registration data. A similar procedure was followed for deaths outside hospitals. The sum of these two distributions yielded an adjusted (for misclassification errors) national estimate of cause-specific proportionate mortality by age and sex.

This proportionate mortality distribution was then fitted to the adjusted mortality envelope to yield the estimated number of deaths by age, sex, and cause for Thailand in 2005. This assumes that the extent of underreporting is similar for deaths that occur inside and outside of hospitals. Simulations of plausible differential undercounts of mortality from the two sources (in-hospital and out-of-hospital deaths) had very little effect on the adjusted age-specific death rates (results not shown). These estimates were then used to describe the rank order and magnitude of leading causes of death in Thailand by sex and age.

We then corrected these adjusted estimates of mortality by redistributing ill-defined deaths. Four specific corrections were made to redistribute ill-defined causes. First, ill-defined causes from chapter R of ICD-10 (20,824 deaths in the corrected VR, 94% of which occurred at home) were proportionately redistributed across all causes apart from injuries. Second, injury deaths with a code of undetermined intent (256 deaths in the corrected VR, half having occurred at home) were proportionately redistributed across all other injury categories. Third, cancer deaths with an unspecified location (1,909 deaths, 65% of which occurred at home) were proportionately redistributed across 13 of the 17 cancer categories but not to cancers of the lung, liver, ovary, and pancreas, which were identified in US vital statistics data not to contribute significantly to ill-defined cancer coding [13]. Fourth, a proportion of ill-defined cardiovascular codes (including codes for heart failure, cardiac arrest, and atherosclerosis, 7,284 deaths, 79% of which occurred at home) were redistributed to ischemic heart disease and the rest to the category of other cardiovascular disease using correction factors from the Global Burden of Disease study comparing proportions of nonstroke cardiovascular deaths coded to IHD and ill-defined cardiovascular disease codes between countries [13].

## Results

### Levels of mortality

Life tables, by sex, are a convenient way to describe age-specific death rates in a population. Table S1 [see Additional file 1] shows abridged life tables for males and females in Thailand in 2005 based on the adjustments described earlier. Key life table parameters are summarized in Table 1 in terms of life expectancy at birth (e_o_), probability of child death before age 5 (_5_*q*_o_), and a measure of adult mortality, namely the probability of dying at ages 15 to 59 (_45_*q*_15_). For comparison, the raw or unadjusted values of these parameters are also shown.

**Table 1:** Mortality and life table parameters from registration data, and after adjusting for incompleteness of registration, Thailand, 2005.

Parameter	Males		Females	
	
	Registration	Final estimate	Registration	Final estimate
e_0 _(years)	70.78	68.53	77.77	75.63
_5_q_0_	0.012	0.022	0.010	0.019
_45_q_15_	0.220	0.244	0.108	0.125
Total deaths	225,622	251,671	169,752	190,499

Our adjustments result in a lower life expectancy at birth than the registration data suggest, by about two years for both males and females. The estimated risk of adult mortality (between ages 15 and 60 years) in males is 24%, double that for females (12%), irrespective of whether adjusted or unadjusted data are used. These levels of mortality in turn are about double those prevailing in some developed countries, including Australia [14]. Overall, just more than 447,000 deaths were estimated to have occurred in Thailand in 2005, about 47,000 or 11.8% more than were registered.

### Leading causes of death

**Figure 1:**
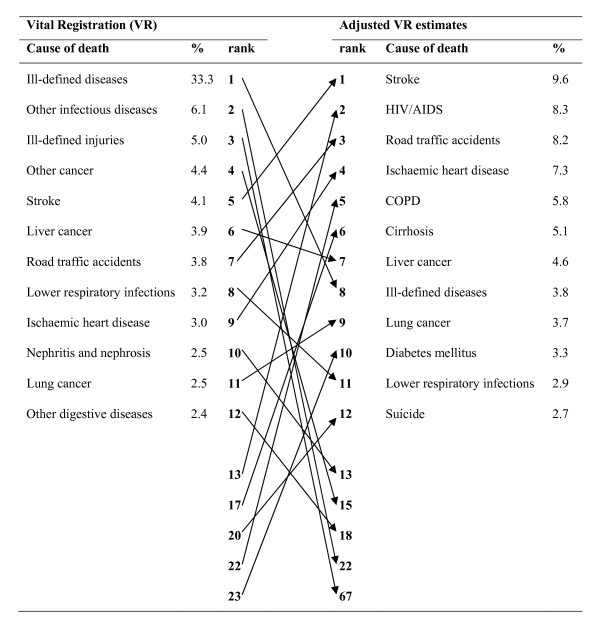
**Adjusted cause-specific mortality fractions compared with vital registration data, Thailand, 2005, males**.

**Figure 2:**
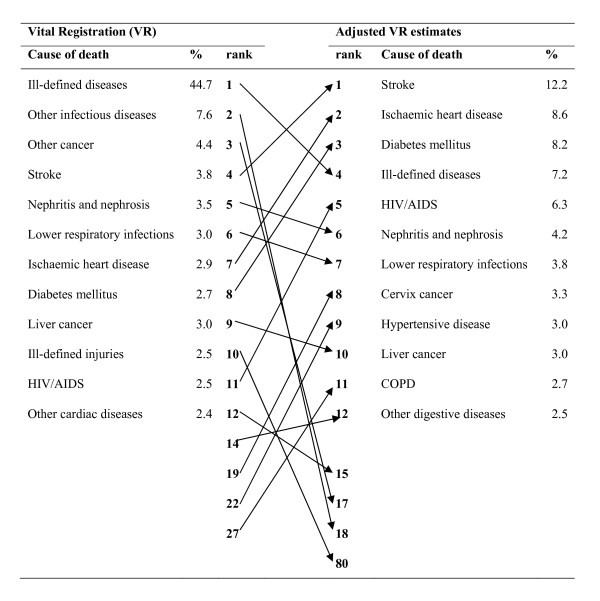
**Adjusted cause-specific mortality fractions compared with vital registration data, Thailand, 2005, females**.

The corrected vital registration estimates of deaths from specific causes from our study are remarkably different in both rank order and magnitude from what is observed in the registration data for both males and females (Figures 1 and 2). Our findings suggest that stroke was the leading cause of death in Thailand, causing 10.7% (about 48,000) of deaths in 2005. Stroke as a cause of death far exceeds the next ranked causes, ischemic heart disease and HIV/AIDS, estimated to cause 7.8% and 7.5% of deaths in 2005, respectively. Road traffic accidents and diabetes each caused more than 20,000 deaths. Full details of the vital registration adjusted numbers of deaths by five-year age group, sex, and cause are given in Table S3 [see Additional file 1]. These are substantially different numbers of deaths to what is recorded in vital registration and have important implications for Thai national disease and injury control programs, including targets, which have been developed on the basis of much lower, and incorrect, cause-of-death numbers.

A more accurate understanding of the rank and magnitude of mortality from specific conditions is of critical importance for health policy and planning in Thailand, as elsewhere. Among males, proportionate mortality from stroke, road traffic accidents, and ischemic heart disease is more than double what is observed from death registration. Those three causes along with HIV and COPD comprise the top five causes of death. Neither HIV/AIDS nor COPD were ranked in the top 10 causes of male deaths based on vital registration. Suicide, diabetes, and cirrhosis of the liver were also among the leading causes of male deaths in Thailand, but were insignificant in vital registration. The considerable changes in rankings for these various causes of death are evident from the steep slopes of most of the corresponding arrows in Figure 1. The fraction of ill-defined diseases among all deaths, for example, fell tenfold. Other vague diagnoses such as "other malignant neoplasms" or "ill-defined injuries" (uncertain whether accidently or purposefully inflicted) declined substantially as causes of death.

Similar changes can be observed in the cause-of-death profile for females. Our findings suggest that deaths from diabetes among women are vastly underappreciated in Thailand, as is cervical cancer, rising to the second and eighth leading causes, respectively. Hypertensive diseases and COPD also appear among the leading causes of death among females in Thailand but are not identified in the top 20 causes from vital registration. As observed in the field studies, these changes are primarily due to the reclassification of deaths from ill-defined and nonspecific "other" categories in the vital registration.

### Leading causes of death by age

For prevention, it is of greater importance to correctly identify the leading causes of premature death. Figures 1 and 2 have contrasted the leading causes of death in corrected vital registration, based on our research methods, with what is recorded in the Thai vital registration. Important differences with significant policy and program implications have been observed. However, at some ages, a substantial fraction of deaths is still coded to ill-defined and vague causes of death, given the limitations of verbal autopsy methods. We have applied the techniques developed for the Global Burden of Disease Study [13] to redistribute these deaths and thus maximize the utility of the cause-of-death estimates for policy. These adjusted (for incompleteness and misclassification) and corrected (for poorly specified certification and coding) estimates of causes of death in Thailand are what we consider the best complete specification of mortality by age, sex, and cause in Thailand. They are shown in full age, sex, and cause detail in Table S4 [see Additional file 1] and summarized in Tables 2, 3, 4 and 5.

**Table 2:** Leading causes of death at ages below 15 years, Thailand, 2005.

Cause of death	% of deaths
Birth asphyxia and birth trauma	12.7
Road traffic accidents	11.6
Drowning	10.0
Low birth weight	9.6
Congenital heart anomalies	6.0
HIV/AIDS	4.1
Anencephaly	3.7
Down syndrome	3.2
Leukaemia	1.6
Other causes	37.3
Total (N)	22,103
	
Ill-defined*	*0.6*

* Redistributed across specific causes

**Table 3:** Leading causes of death at ages 15-49 years, Thailand, 2005.

**Males**		**Females**	
	
**Cause of death**	**% of deaths**	**Cause of death**	**% of deaths**
	
HIV/AIDS	22.6	HIV/AIDS	30.7
Road traffic accidents	19.0	Road traffic accidents	6.5
Cirrhosis of the liver	6.6	Cervix cancer	5.8
Suicide	5.7	Stroke	4.1
Homicide	4.8	Diabetes mellitus	3.6
Stroke	3.9	Suicide	3.3
Liver cancer	3.2	Cirrhosis of the liver	3.0
Ischaemic heart disease	2.7	Liver cancer	2.9
Drowning	2.3	Breast cancer	2.8
Lower respiratory infections	1.9	Nephritis and nephrosis	2.5
Other causes	27.3	Other causes	34.9
Total (N)	83,419	Total (N)	36,503
			
Ill-defined*	*1.7*	Ill-defined*	*0.3*

* Redistributed across specific cause

**Table 4:** Leading causes of death at ages 50-74 years, Thailand, 2005.

**Males**		**Females**	
	
**Cause of death**	**% of deaths**	**Cause of death**	**% of deaths**
	
Stroke	12.3	Diabetes mellitus	12.3
Ischaemic heart disease	11.1	Stroke	10.5
Liver cancer	7.5	Ischaemic heart disease	9.6
COPD	6.8	Nephritis and nephrosis	5.4
Lung cancer	6.0	Cervix cancer	4.8
Cirrhosis of the liver	5.7	Liver cancer	4.8
Diabetes mellitus	5.4	Lung cancer	3.7
Road traffic accidents	3.1	COPD	3.6
Nephritis and nephrosis	2.9	Hypertensive disease	3.6
Lower respiratory infections	2.5	Cirrhosis of the liver	3.3
Other causes	36.8	Other causes	38.5
Total (N)	98,602	Total (N)	71,902
			
Ill-defined*	*2.5*	Ill-defined*	*2.3*

* Redistributed across specific causes

**Table 5:** Leading causes of death at ages 75 years and over, Thailand, 2005.

**Males**		**Females**	
	
**Cause of death**	**% of deaths**	**Cause of death**	**% of deaths**
	
Stroke	15.1	Stroke	19.4
COPD	12.5	Ischaemic heart disease	12.1
Ischaemic heart disease	8.8	Diabetes mellitus	7.3
Lower respiratory infections	5.4	Lower respiratory infections	7.2
Lung cancer	3.8	Nephritis and nephrosis	4.3
Diabetes mellitus	3.7	Hypertensive disease	4.1
Nephritis and nephrosis	3.6	COPD	3.5
Hypertensive disease	3.3	Diarrhoeal diseases	2.6
Cirrhosis of the liver	3.2	Falls	2.6
Tuberculosis	2.8	Liver cancer	1.8
Other causes	37.8	Other causes	35.3
Total (N)	60,146	Total (N)	74,429
			
Ill-defined*	9.4	Ill-defined*	15.2

* Redistributed across specific causes

Among children, perinatal conditions and congenital anomalies dominate the cause-specific mortality structure (Table 2), as might be expected in a population with levels of under-5 mortality around 20 per 1000 [8]If true, this would suggest that there has been considerable progress in the control of childhood mortality from infectious diseases (diarrhea, pneumonia, and vaccine-preventable diseases) in Thailand, including HIV/AIDS, a reflection of the very successful program in Thailand to prevent mother-to-child transmission [15].

At ages 15-49 years, HIV/AIDS is the leading cause of death in both males and females, and, with the exception of road traffic deaths among males, is by far the principal concern for preventing mortality at young adult ages (Table 3). Overall, more than one-third (37%) of all male deaths at these ages are due to injuries. This is even higher than the fraction recorded in vital statistics (33%) and requires urgent policy action. The observation that cervical and breast cancer are among the leading causes of death for women at these ages should also guide the development and implementation of screening, diagnostic, and therapeutic intervention programs.

The cause-specific mortality patterns at older adult ages, 50-74 years and 75 years and above, are relatively similar for males and females (Tables 4 and 5). Cardiovascular diseases, chronic lung disease, and cancers cause the majority of deaths at these ages, as elsewhere [8]. Mortality from diabetes, particularly among women, is noteworthy, causing one in eight female deaths at ages 50-74 years. The presence of liver, kidney, and respiratory diseases among the leading causes of death in both adult men and women suggests the need for better primary prevention, as well as early diagnoses and treatment programs for these conditions, particularly in middle age.

## Discussion

The absence of reliable cause-of-death statistics from vital registration systems in countries such as Thailand necessitates extensive demographic and epidemiological adjustment to reported data in order to derive plausible estimates of mortality by age, sex, and cause for health policy and periodic burden of disease assessment. Well-designed field research is far preferable to generate the empirical evidence necessary for making adjustments to imperfect registration data than basing these adjustments on fragmentary epidemiological evidence or cause-of-death models. The research reported in this paper and elsewhere [4,5,7] provides an example of the design, conduct, and utility of field studies in generating empirical evidence to derive useful estimates of cause-specific mortality by age and sex for Thailand in 2005. The empirical evidence is contained in Table S5 [see Additional file 1], which provides the detailed ICD code for the underlying cause for all 11,984 deaths derived from each source as available; i.e., vital registration (VR); medical records review (MR); and/or verbal autopsy (VA). Although not a substitute for sustained development of vital statistics systems, studies of the type we have undertaken allow maximum public health benefit to be gained from poorly functioning vital registration systems by enabling the estimation of likely prevailing levels of mortality and causes of death from local evidence. In the Thai context, the key strengths of this study lie as much in the development of capacity for routine application of novel, alternative data collection methods as in the final description of likely mortality patterns prevailing in the country.

A comparative analysis of findings from this study with findings from a study with similar objectives conducted in 1999 [16] reveal several important differences (Table 6). The most notable is the very substantial decline in mortality from HIV/AIDS, especially among males (1999: 17% of deaths - rank 1; 2005: 8% of deaths - rank 2) and to a lesser extent among females (1999: 8% of deaths - rank 2; 2005: 6% of deaths - rank 4). This decline mirrors the very significant decline in HIV prevalence that has been observed since the mid-1990s, as well as the widespread introduction of antiretroviral therapy [17,18]. After all adjustments and redistributions, stroke remained the leading cause of death for Thai females and became the leading cause for males, replacing HIV/AIDS. Ischemic heart disease, after adjustments, is among the leading causes of death for both sexes, as is diabetes for females and road traffic accidents for males. The predominance of major vascular diseases as leading causes of death in Thailand is consistent with risk factor trends. Overweight and obesity is rising (currently 30% of adults 15 years and over), and prevalence of hypertension is 50% in some age groups [19,20].

**Table 6:** Leading causes of death (all ages) as a proportion of all deaths in 1999 and 2005, Thai Burden of Disease studies.

**Males**	**2005**	**1999**	**Females**	**2005**	**1999**
	
**Cause**	**%**	**%**	**Cause**	**%**	**%**
	
Stroke	9.6	9.0	Stroke	12.2	15.0
HIV/AIDS	8.3	16.5	Ischaemic heart disease	8.6	5.6
Road traffic accidents	8.2	8.7	Diabetes mellitus	8.2	7.8
Ischaemic heart disease	7.3	5.0	HIV/AIDS	6.3	8.4
COPD	5.8	5.2	Nephritis and nephrosis	4.2	2.6
Cirrhosis of the liver	5.1	2.8	Lower respiratory infections	3.9	3.1
Liver cancer	4.6	7.2	Cervix cancer	3.3	2.0
Lung cancer	3.7	3.3	Hypertensive disease	3.0	1.1
Diabetes mellitus	3.3	3.1	Liver cancer	3.0	5.0
Lower respiratory infections	2.9	2.1	COPD	2.7	3.3
Suicide	2.7	2.8	Road traffic accidents	2.4	2.8
Nephritis and nephrosis	2.4	1.6	Cirrhosis of the liver	2.3	1.7

Overall, the composition of the top 15 causes of death has remained relatively stable over the six-year period, although the rankings of specific causes may have changed. Interestingly, proportionate mortality from major liver diseases (liver cancer, cirrhosis of the liver) has remained relatively constant (9% to10% for males, 5% to 6% for females) but with a sharp increase in cirrhosis mortality and a comparable decrease in liver cancer. This may be largely due to better diagnostic practices in recent years.

These findings suggest that small but discernable time trends in cause-specific mortality can be observed from epidemiological evaluation studies conducted periodically, which can theoretically provide critical evidence for program evaluation as well as priorities for health policy. While such trend analyses are undoubtedly useful, caution in their interpretation is required due to differences in methods and in study design and implementation. Perhaps more importantly, mortality estimates based on the procedures that we have applied would be more interpretable for policy if they were accompanied by uncertainty estimates, reflecting largely the uncertainties inherent in the VA instrument as well as sampling errors. Further research to better quantify uncertainty in VA-based estimates of mortality is a priority if studies such as ours are to be more informative for policy debates.

Despite the potential value of these findings, a number of limitations of the study need to be kept in mind. First, the completeness of mortality registration in Thailand remains uncertain, and while this uncertainty is unlikely to be large, important biases could result from our lack of understanding about the true extent of mortality undercounting in different population subgroups, such as potential differences in completeness between home and facility deaths. A further limitation is the potential biases arising from the sampling design of the field verification studies [4]. There is also some uncertainty about the generalizability of our study findings, arising from the different cause-of-death ascertainment procedures that were used for deaths in hospitals and outside hospitals [5,7]. For deaths outside hospitals, there are methodological constraints associated with the use of verbal autopsy methods because in most countries where they would be applied, including Thailand, medical records for deaths that occur at home are too poor to permit competent validation studies. Even the process of medical records review that we have employed, and that has been widely used in VA validation studies, is at best an approximation to the gold standard that might be expected from autopsy [21-23]. While these and other possible limitations contribute to the uncertainty of mortality estimates derived from this research, we believe that we have vastly decreased the uncertainty around registered mortality data in Thailand and thus greatly increased their utility for epidemiological assessments, priority setting, and monitoring of specific health conditions.

Table 5 shows that ill-defined conditions still account for 10% to 15% of deaths among the elderly, even after application of our methods. This suggests that while our study has substantially improved the categorical attribution of causes of death, the absence of medical certification for deaths occurring outside of hospitals will continue to hinder the development of accurate cause-of-death statistics at older ages. Adequate record linkage between death registration and hospital medical records might improve this situation, if implemented within the legal, administrative, and societal context of civil registration and vital statistics systems in Thailand. There are, however, many instances among the elderly where death occurs suddenly, with no apparent disease. In such cases, virtually no symptoms or useful clinical information will be gained from the VA interview, resulting in a VA diagnosis of senility or some other ill-defined code. This is inevitable, and as long as this fraction can be kept to a reasonable minimum, with the vast majority of such deaths confined to the oldest ages, VA procedures can, if implemented rigorously, identify causes of death of policy relevance.

The frequent monitoring of birth and death registration completeness in Thailand, both by government and academic institutions, has undoubtedly been an important factor in achieving better levels of vital statistics completeness. The collaboration of the Thai Ministry of Public Health with researchers involved in this study indicates that government clearly understands that in order to fully benefit from the significant resource allocation that Thailand makes each year to maintain its vital registration system, it must ensure that the data collected are sufficiently reliable for planning purposes. As the completeness of birth and death registration in Thailand approaches 100%, the focus will increasingly be on improving the quality of cause-of-death information to more reliably support health policies and programs.

There are several obvious lessons that can be drawn from this research. The study, conducted over the period 2004-2008, has had an important capacity-building component that will aid sustainability. As a result, extensive local capacity now exists for conducting verbal autopsy and for physicians to more reliably certify the cause of death from information collected at interview. This was accomplished by employing and training local government health staff as VA interviewers and supervisors for field data collection and training provincial physicians in the correct procedures for medical certification of cause of death from VA.

Importantly, the Thai government has already acted on our study findings. An independent death certification audit has been carried out by national experts based on ICD-10 principles to identify common errors and misunderstandings that have a material impact on the death certification process [Pao-in W. Report on death certificate audit project in Thailand, 2008-2009. Unpublished]. An interesting finding from this study is that the physicians who frequently misclassified the underlying cause of death were those younger than 30 years of age and older than 50 years of age (in other words, those who had recently graduated and were too busy or inexperienced, or older physicians who had never learned how to certify deaths correctly). One potential approach to improving cause-of-death certification in these subgroups might be to advocate that the Thai Medical Council include certification and ICD-10 coding in the accreditation curriculum. A system to ensure continuous quality control of death certification in hospitals is also being introduced.

The research has also led to a feasibility study being conducted to reform the routine death registration system. Different mechanisms to strengthen the routine reporting of nonhospital deaths are being investigated. One procedure being tested, for example, is the possibility of health personnel applying a Thai-specific VA instrument based on our methods to more reliably and routinely deduce the cause of all nonmedically certified deaths. Another option under review is the use of statistical algorithms such as the 'symptom pattern' method to infer diagnoses from the pattern of responses on the VA questionnaire, thus obviating the need for physician input [24].

While the cause-of-death system is being reformed, nationally representative cross-sectional evaluation studies such as the one summarized in this paper and elsewhere [4,5,7] might be carried out at five-year intervals to more reliably ascertain the likely causes of registered deaths using a Thai-specific VA instrument. This would ensure that the cause-of-death correction factors now available from our research would be periodically adjusted to reflect health information system developments and provide useful national estimates of causes of death by age and sex, without which any burden of disease assessment is likely to be uninformative.

## Competing interests

The authors declare that they have no competing interests.

## Authors' contributions

YP, CR, TV, TA, JP, and WP contributed to data analysis. ADL and CR drafted the manuscript. YP contributed to the policy implications section. ADL and YP finalized the manuscript. All authors read and approved the final version.

## Supplementary Material

Additional file 1 **Estimated causes of death in Thailand, 2005: Supplemental tables**. Table S1: Estimated abridged life tables for males and females in Thailand, 2005. Table S2: Data on deaths by age, sex and cause from the vital registration system in Thailand, 2005. Table S3: Mortality estimates by age, sex and cause in Thailand, 2005; derived from adjustments to vital registration data based on field studies. Table S4: Mortality estimates by age, sex and cause in Thailand, 2005; based on corrections for completeness of death registration and adjustments to cause of death distributions using the Global Burden of Disease study methods. Table S5: Detailed ICD codes from each data source for deaths included in the field study sample. Click here for file
